# Cognitive control, attention, and the other race effect in memory

**DOI:** 10.1371/journal.pone.0173579

**Published:** 2017-03-10

**Authors:** Thackery I. Brown, Melina R. Uncapher, Tiffany E. Chow, Jennifer L. Eberhardt, Anthony D. Wagner

**Affiliations:** 1 Department of Psychology, Stanford University, Stanford, California, United States of America; 2 Department of Neurology, University of California, San Francisco, San Francisco, California, United States of America; 3 Department of Psychology, University of California, Los Angeles, Los Angeles, California, United States of America; 4 Neurosciences Program, Stanford University, Stanford, California, United States of America; University of Bologna, ITALY

## Abstract

People are better at remembering faces from their own race than other races–a phenomenon with significant societal implications. This Other Race Effect (ORE) in memory could arise from different attentional allocation to, and cognitive control over, same- and other-race faces during encoding. Deeper or more differentiated processing of same-race faces could yield more robust representations of same- vs. other-race faces that could support better recognition memory. Conversely, to the extent that other-race faces may be characterized by lower perceptual expertise, attention and cognitive control may be more important for successful encoding of robust, distinct representations of these stimuli. We tested a mechanistic model in which successful encoding of same- and other-race faces, indexed by subsequent memory performance, is differentially predicted by (a) engagement of frontoparietal networks subserving top-down attention and cognitive control, and (b) interactions between frontoparietal networks and fusiform cortex face processing. European American (EA) and African American (AA) participants underwent fMRI while intentionally encoding EA and AA faces, and ~24 hrs later performed an “old/new” recognition memory task. Univariate analyses revealed greater engagement of frontoparietal top-down attention and cognitive control networks during encoding for same- vs. other-race faces, stemming particularly from a failure to engage the cognitive control network during processing of other-race faces that were subsequently forgotten. Psychophysiological interaction (PPI) analyses further revealed that OREs were characterized by greater functional interaction between medial intraparietal sulcus, a component of the top-down attention network, and fusiform cortex during same- than other-race face encoding. Together, these results suggest that group-based face memory biases at least partially stem from differential allocation of cognitive control and top-down attention during encoding, such that same-race memory benefits from elevated top-down attentional engagement with face processing regions; conversely, reduced recruitment of cognitive control circuitry appears more predictive of memory failure when encoding out-group faces.

## Introduction

Understanding the neural mechanisms that underlie and influence relations between different social groups is of central importance for society. Research has long suggested that social category information, such as race and gender, is automatically encoded [1,2], and that social categories can influence cognition, with implications for behavior in social and legal settings. For example, faces from one’s own racial “group” vs. from another group may be processed differently during perception, which can influence subsequent memory. Same- vs. other-race status affects the perceptual processing of faces within a few hundred milliseconds [3,4]. Moreover, people exhibit a tendency towards more rapid and accurate categorization of other-race faces as being of another race than same-race faces as being of one’s own race [5–11]. This rapid out-group categorization benefit may, paradoxically, relate to a bias for people to engage in deeper, more computationally expensive, in-group processing [6]. As such, group categorization effects may contribute “downstream” to a long-established memory phenomenon known as the Other Race Effect (ORE) [12–14], characterized by better recognition memory for same-race than other-race faces.

OREs in memory have been associated with greater activity in face-sensitive areas of fusiform cortex during the processing of same- than other-race faces [15–17]. Such differences in fusiform processing may reflect this region’s sensitivity to the perceptual expertise that may arise from being exposed to more same-race faces [15,18,19]. However, cognitive [20,21] and attentional modulation during perception, which influences the magnitude of fusiform activity [22,23], may also contribute to OREs in neural activity and memory behavior [15,24]. Top-down attention (directing attention to goal-relevant information) and cognitive control (flexibly aligning cognitive and sensorimotor operations based on processing goals) are tightly coupled sets of processes that can influence what information we encode and in how much detail [25–31]. Top-down attention to, and cognitive control over, face processing could enhance activity in representational cortical areas including fusiform cortex. Such top-down modulatory control could give rise to higher fidelity and/or more differentiated representations of same-race faces, which ultimately could support better recognition memory. Conversely, failure to encode other-race faces could stem from a failure to engage these top-down mechanisms, due to a more superficial level of processing that may interact with potentially more limited perceptual expertise. Using a subsequent memory approach [30,32–34], the present study targeted this mechanistic framing, testing whether the ORE is predicted by differential engagement of attention and cognitive control networks, and their interaction with face-sensitive ventrotemporal cortex, during the encoding of same- and other-race faces.

A growing body of evidence supports the potential contributions of top-down attention and cognitive control processes to group-based biases in memory. Recent imaging studies suggest that race effects in fusiform cortex reflect more than expertise with same-race faces; rather, cognitive factors and processing goals can exert a powerful influence over how same- vs. other-race faces are processed [20,21]. Specifically, when the race of face stimuli is orthogonalized to alternative, task-relevant group categories (e.g., sports teams), activation differences in fusiform cortex shift from same-*race* to same-*group* [35–37]. Such shifts are also observed in behavioral measures of automatic face preference, valence evaluations, and in memory biases [36,38]. Such data suggest that arbitrary and experimentally induced group membership categories can result in changes in how attention and control processes are allocated during face processing.

Top-down attention and cognitive control are supported, in part, by two large-scale frontoparietal networks [39–43] that respectively include (a) the superior frontal sulcus (SFS), superior parietal lobule (SPL), and medial intraparietal sulcus (IPS), and (b) the inferior frontal sulcus (IFS) and lateral IPS. These cortical networks are associated with distinct, non-overlapping resting-state connectivity profiles in fMRI [44], which enables hypothesis testing about their respective contributions to cognitive and mnemonic functions. How we coordinate cognitive and sensorimotor operations and direct our attention according to processing goals has a direct impact on what information we encode. Consequently, recruitment of these two frontoparietal networks is well established to influence episodic encoding success for a variety of stimuli including faces, as indexed by subsequent memory performance [25–30].

To the extent that attention is differentially allocated to same- vs. other-race faces, this predicts that frontoparietal components of the top-down attention network should be differentially active during their encoding, with activity relating to later memory. Conversely, or in addition, reduced engagement of the cognitive control network for other-race faces may be predictive of memory failure, reflecting a case where encoding of faces from another race is subjected to a more superficial level of processing. Such a hypothesized relationship could be attributed to decreased motivational relevance for encoding differentiated representations of individuals with whom we do not share social categories [37,38], and the relationship of control processes to encoding success could further interact with reduced perceptual expertise for faces from other racial groups. To examine the contribution of differential engagement of frontoparietal attention and control networks to the ORE, we combined fMRI with an elaborative intentional encoding task, and quantified differences in the engagement of top-down attention and cognitive control circuitry during same- and other-race face encoding. Leveraging functional connectivity analyses, we further tested whether interactions between top-down attention and cognitive control networks, and fusiform cortex are more strongly predictive of memory success for same-race faces. Results provide novel evidence that failure to encode other-race faces is characterized by reduced engagement of parietal cognitive control resources, and fusiform processing of same-race faces is characterized by greater interaction with parietal top-down attention resources.

## Materials and methods

### Participants

Nineteen healthy, right-handed male participants were recruited from Stanford University and its surrounding communities. We restricted the participant pool to males in order to minimize potential contributions of gender to the targeted same-/other-race effects. Participants were 18–31 yrs old (mean ± SD: 23.26 ± 4.69yrs) native English speakers with no history of neurological complications, and were either African American (AA; *n* = 5) or European American (EA; *n* = 14) according to self-report. Our sample size was based on typical participant counts for within-participants studies in the field [15,37,45]. Because our sampling procedure did not target comparisons between participant subgroups, and because additional participants collected had to be excluded from analysis (see below), the analyses reported here focus on within-participants statistics collapsing across the AA and EA participant subgroups.

Because the present analyses focus on subsequent memory effects during encoding, data from six additional participants were collected but omitted from analyses because of inadequate trial counts (fewer than 6 trials in a subsequent memory bin [remembered or forgotten]; n = 4) or *d′*s that were near chance (overall *d’* = −0.08, n = 1; same-race *d’* = 0.11, n = 1). One additional participant withdrew from scanning before completing the experiment. Participants were compensated $20/hr for their involvement, and gave written informed consent, in accordance with Stanford University Institutional Review Board approved procedures for this study. The present study was conducted over the course of 2015–2016, with initial data collection in 2012.

### Stimuli

Stimuli consisted of 400 color photographs of male faces (200 AA, 200 EA), shared from the databases of J. Eberhardt and G. Golarai. Face stimuli were standardized for neutral facial expression and background illumination, and included head and neck only. Face stimuli were presented with a white background against a gray screen and with a black central fixation crosshair. For each participant, face stimuli were divided into two sets using stratified random sampling by race to assign stimuli to be studied during the encoding phase (OLD items; 100 AA, 100 EA) or to serve as foil items at retrieval (NEW items; 100 AA, 100 EA).

### Day 1: Encoding

The experiment included a face encoding scan session, followed ~24 hr later by a subsequent recognition memory session. On Day 1, participants were scanned while intentionally encoding 200 male faces (100 AA faces and 100 EA faces). Participants were instructed to use an elaborative encoding strategy to memorize the stimuli, whereby they were to generate imaginative stories involving the individuals pictured in the stimuli in a manner that would facilitate their later retrieval of the faces the following day. The use of an intentional encoding paradigm [15] targeted the notion that in our daily lives we frequently intentionally try to encode the identity of individuals we encounter, in order to facilitate future identification and social interaction. To confirm that participants were attending to stimuli and engaging in the task, they were instructed to press a button under their right index finger when each face appeared.

**Fig 1:**
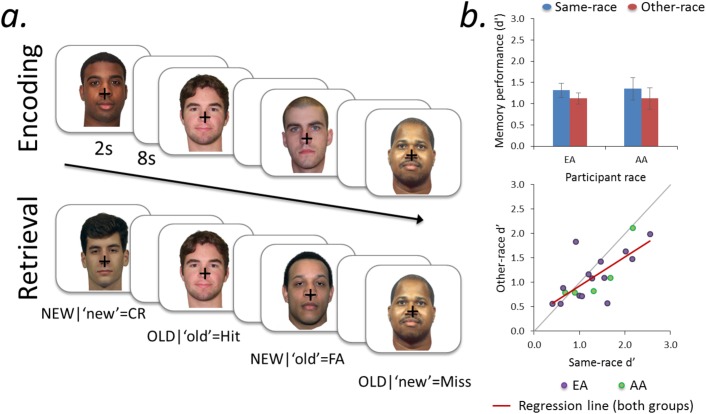
Task design and behavioral performance. a) Representative stimuli and trial structure for encoding and subsequent memory (retrieval) task phases, separated by ~24 hr. b) Top graph: subsequent memory performance for same-race and other-race faces, by participant race. Bottom graph: distribution of same-race and other-race subsequent memory performance (*d’*), by participant race (purple and green points). Same-race and other-race d’ were strongly correlated (*r* = 0.72), and participants with greater same-race performance tended to demonstrate larger Other Race Effects (same-race–other-race d’; i.e., slope of the regression line (red line) < 1). AA = African American; EA = European American; Error bars reflect group SEM.

Each face was presented for 2 s, with an 8 s interstimulus interval (ISI) for a total of 10 s per trial (Fig 1A). Stimuli appeared in a pseudorandom order such that no more than three presentations of the same race appeared consecutively. After the full set of 200 stimuli was presented, the same faces were presented for a second repetition in a different order. Participants were informed that this second repetition was intended as a second study opportunity, to help them boost their encoding of the faces. Stimuli were presented across eight runs, with 50 faces per run. The first four runs consisted of first presentations of the stimuli, and the second four runs consisted of second presentations of the stimuli. The analyses reported herein focus on the relationship between activity during the first encoding experience and subsequent memory performance during the retrieval phase.

### Day 2: Retrieval

Approximately 24 hr later, participants returned for a scanned retrieval phase. In this phase, participants were presented with all 200 studied faces, interspersed pseudorandomly with 200 novel faces, and were asked to provide an “old/new” recognition response on each trial. As in the encoding phase, each face was presented for 2 s with an 8 s ISI, for a total of 10 s per trial. Participants were given the entire trial length (10 s) to respond to each face but were instructed that both speed and accuracy were important, with the latter emphasized to ensure adequate behavioral performance. Stimuli were presented across eight scanning runs. Runs were split into two tasks, with the first four runs comprising the old/new recognition task (termed the “explicit memory” task) that is of interest here (note: the second four runs consisted of a “concealed memory” task, which was included for a complementary study targeting the effects of effortful memory concealment on the neural correlates of retrieval [46]).

We focus on the behavioral data from the explicit memory task, which provide subsequent memory outcomes that were used to analyze the encoding-phase fMRI data. The explicit memory task contained 50 OLD AA faces, 50 OLD EA faces, 50 NEW AA faces, and 50 NEW EA faces. Stimuli were balanced for race within each run and presented pseudorandomly such that no more than three presentations of the same race (AA or EA) or condition (OLD or NEW) appeared consecutively. In the explicit memory task, participants made old/new recognition memory judgments: they pressed a button with their (1) index finger if they judged a stimulus to be “old,” i.e., previously encountered during the encoding session, or (2) middle finger if they judged the face to be unstudied, or “new.” Alternating across odd/even runs, participants switched which hand they used to make the old/new judgments.

### Functional localizer task

After retrieval, participants performed two runs of a block-design functional localizer task to enable identification of face-sensitive fusiform cortex [47]. The localizer was used to ensure that seed regions in fusiform cortex that were defined based on univariate subsequent memory analysis and submitted to PPI analysis (see below) also co-localized to “face-sensitive” cortex using independent data.

In the localizer, photographs were presented of intact AA faces, intact EA faces, scrambled AA faces [wherein facial features (eyes, nose, and mouth) were rearranged within the face], scrambled EA faces, scenes, abstract objects, and body parts. Each image was presented for 0.8 s with a 0.2 s ISI in 12 s blocks. Each run consisted of two blocks of each condition presented in pseudorandom order, interspersed with four blank blocks, wherein participants passively viewed the fixation cross. Participants were instructed to respond when two consecutive images were identical. Each block of 12 items contained 0, 1, or 2 repeated items.

### MRI data acquisition

Whole-brain imaging was performed with a 3.0 T GE (Discovery MR750) MR scanner. A T2-weighted anatomical volume was collected immediately before the functional runs, using a flow-compensated spin-echo pulse sequence, and a T1-weighted whole-brain spoiled gradient recalled high-resolution (SPGR) anatomical image was collected at the end of Day 1 (encoding). Each functional volume, collected with a T2*-weighted echo planar imaging (EPI) pulse sequence, consisted of 36 slices acquired in an interleaved ascending progression, parallel to the anterior commissure–posterior commissure plane. Functional volumes were collected (64 × 64 matrix) using a repetition time (TR) of 2 s, echo time of 30 ms, and a field of view of 21 cm. In-plane resolution was 3.28 mm^2^, and slice thickness was 3.3 mm. A total of 260 volumes were collected for each of the four explicit memory encoding runs on Day 1, with the initial four volumes of each run discarded to allow for T1 equilibration.

### Univariate fMRI analyses

Statistical Parametric Mapping (SPM8; Wellcome Department of Cognitive Neurology, London, UK; http://www.fil.ion.ucl.ac.uk/spm/software/spm8), run in MATLAB 7.13 (R2011b; MathWorks), was used for data preprocessing and univariate and PPI (see below) analyses. Standard preprocessing procedures were applied to the data. Functional volumes were slice-time corrected to the middle slice in time, motion corrected, spatially realigned to the first volume, and then realigned to the mean volume of the session. The T2-weighted anatomical volume from the Day 2 (retrieval) session was coregistered to the mean functional volume, the T1-weighted anatomical volume was then coregistered to this coregistered T2-weighted volume, and then the T1-weighted volume was segmented into gray matter, white matter, and CSF, with the resulting images normalized to templates in Montreal Neurological Institute (MNI) space. Functional volumes were normalized into standard space based on the transformation parameters obtained during segmentation, and resampled into 4-mm^3^ voxels. All images were then spatially smoothed with an 8-mm full-width at half-maximum (FWHM) Gaussian kernel.

First-level general linear models (GLMs) were computed for each participant by modeling each encoding event based on the race of the stimulus, the retrieval phase task later performed on the stimulus (explicit and concealed memory tasks), and the subsequent memory outcome (hits and misses). Thus, eight principal encoding event regressors were included in the model: AA hits, AA misses, EA hits, and EA misses from the first encoding presentation for each of the two subsequent retrieval tasks. Regressors modeling movement parameters estimated during realignment and session effects were included as nuisance factors. Each encoding event was modeled as a 2-s epoch, corresponding to the duration of the face stimuli, and convolved with the canonical hemodynamic response function. An AR(1) model was used to account for serial autocorrelations. GLM parameters were estimated with classical (restricted maximum likelihood) algorithms. Linear contrasts of the resulting parameter estimates were used to investigate and test effects of interest.

**Fig 2:**
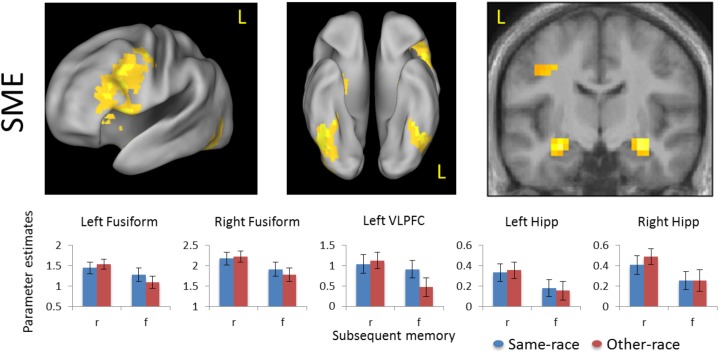
Subsequent memory effects. Activity during the first encoding presentation of each face was predictive of subsequent memory success in regions previously implicated in successful encoding. *Bar graphs*: mean parameter estimates extracted from significant clusters, separately for same- and other-race face encoding trials (Error bars reflect group SEM). Activity is rendered on the 3D Caret inflated cortical surface or the 2D mean across-subject anatomical image, both in standardized MNI space; height and extent thresholds: *p* < 0.005, *k* = 33, FWE *p* < 0.05. r = remembered; f = forgotten; Hipp = hippocampus; VLPFC = ventrolateral prefrontal cortex.

Second-level group analyses focused on subsequent memory and race effects during initial encoding trials that were later tested in the explicit memory task. Group analyses were conducted with contrast images generated at the first level for each participant of remembered>forgotten trials for same-race and other-race faces, with participants treated as a random effect. Voxel-level contrast effects (e.g., Fig 2) were examined, thresholded at *p* < 0.005 within a mask excluding signal susceptibility and ventricles; an extent threshold of 33 contiguous voxels was further applied to maintain a familywise error (FWE) rate of *p* < 0.05, calculated using 10,000 simulations in 3dClustSim (http://afni.nimh.nih.gov/afni/). These statistical criteria were also applied to the voxel-level PPI analysis (described below).

#### Dorsal attention and cognitive control network regions of interest

**Fig 3:**
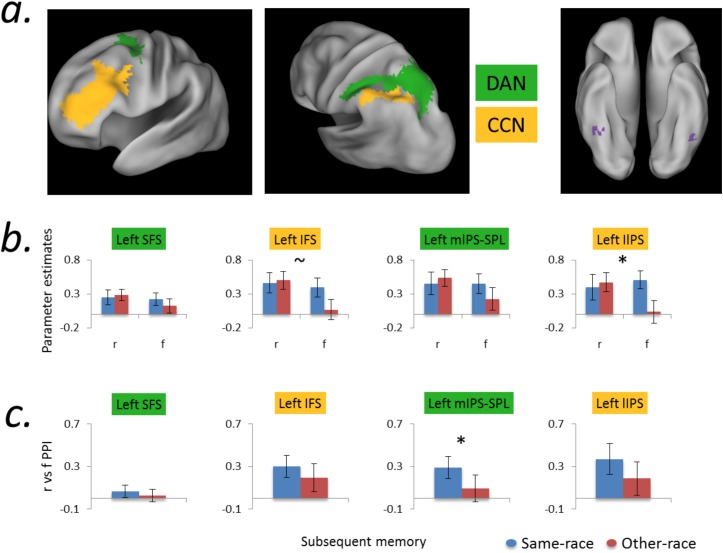
Subsequent Memory Effects as a function of the Other Race Effect. a) Left panels: 3D renderings of dorsal attention network (DAN, green) and cognitive control network (CCN, orange) ROIs. Right panel: Group-level SME “parent” seeds (purple) for PPI analysis (Fig 3c). b) Univariate activity as a function of subsequent memory and same-/other-race face status. There was a tendency towards greater SMEs for other- than same-race faces in left frontoparietal components of the CCN; OREs on the SME were qualitatively similar, but quantitatively reduced, in frontoparietal components of the dorsal attention network. Specifically, the ORE on the SME reached significance in lateral IPS (CCN) and was at trend level in IFS (CCN). c) PPI SME analyses as a function of the ORE. Right fusiform SME connectivity during encoding was greater for same- than other-race faces in the bilateral parietal DAN nodes (right hemisphere not shown); qualitatively similar patterns were observed in the CCN. r = remembered; f = forgotten; SFS = superior frontal sulcus; IFS = inferior frontal sulcus; mIPS-SPL = medial IPS and superior parietal lobule; lIPS = lateral IPS. Error bars reflect group SEM. * = ORE × SME interaction significant at *p*<0.05, ~ = *p*<0.10.

We hypothesized that successful encoding of same- and other-race faces is differentially predicted by engagement of top-down visuospatial attention and cognitive control frontoparietal networks. To test this hypothesis, we adopted a targeted region of interest (ROI) approach for our principal analyses. Specifically, following other recent work [43,48], analyses were constrained to MNI-space ROIs derived from the “7 network” intrinsic functional connectivity cortical parcellations from Yeo and colleagues [44] (https://surfer.nmr.mgh.harvard.edu/fswiki/CorticalParcellation_Yeo2011). Analyses used two frontoparietal ROIs associated with cognitive control (lateral prefrontal cortex, spanning the IFS, and lateral IPS), and two frontoparietal ROIs associated with top-down attention (posterior SFS, which is a putative homolog of the frontal eye fields, and medial IPS-SPL) (Fig 3 –color coding consistent with that in [44]). Note that we masked the mIPS-SPL ROI to exclude voxels along the lateral surface of occipitotemporal cortex (i.e., voxels beyond the bounds of parietal cortex), using the crown of the posterior angular gyrus as the lateral boundary of this ROI. For both univariate and PPI analyses, functional data from these four ROIs were analyzed separately for the left and right hemispheres.

Analyses used a principled means of data reduction to limit the number of pairwise comparisons (*t*-tests), and to evaluate potential differences in the extent to which computations in the top-down / dorsal attention network (DAN) and the cognitive control network (CCN) contribute to OREs in memory. Specifically, univariate subsequent memory effects (remembered>forgotten) and memory-related PPIs from these ROIs were first submitted to separate frontal and parietal cortex repeated-measures ANOVAs in R (R Core Team [2013]. R: A language and environment for statistical computing. R Foundation for Statistical Computing, Vienna, Austria. http://www.R-project.org/), testing for main effects of stimulus race (OREs; differences in subsequent memory effects for same- vs. other-race faces) and interactions between ORE magnitude and network (DAN and CCN).

### Psychophysiological interaction (PPI) analyses

A central question is whether coupling of DAN / CCN regions with face processing regions differs for same- and other-race faces at encoding. To address this question, we conducted a PPI analysis [49] using memory-sensitive bilateral fusiform cortical voxels as functional seeds. This approach enabled a test of the interactions between DAN / CCN and face encoding in fusiform cortex, and a voxel-wise exploration of whether fusiform coupling with the rest of the cortex manifests differently during same- and other-race face encoding. Accordingly, PPIs were computed in SPM8 using fusiform seeds and the psychological factors of subsequent memory success and stimulus race. Resulting participant-specific PPI statistical maps were entered into group-level analysis in SPM to characterize memory-predictive coupling with fusiform cortex, and differences in this coupling related to same- vs. other-race face processing (OREs).

Fusiform seeds were defined in three stages, using a method that did not presuppose any differences in same- and other-race face processing. First, a group-level contrast of encoding activity for all subsequently remembered>subsequently forgotten faces (collapsing across same- and other-race encoding trials) was conducted at a voxelwise threshold of *p*<0.005 (k = 33, FWE *p* < 0.05). Group-level subsequent memory effect peaks were identified in left and right fusiform cortex (“parent” seeds, Fig 3A). Second, the coordinates of these peaks were then shifted to participant-specific local maxima from the analogous 1^st^-level contrast (threshold of *p* < 0.2), with a maximum allowable distance between participant-specific seeds and the group-level peak of less than 16 mm (2*FWHM). The participant-specific seeds were further required to remain in face-sensitive fusiform cortex (defined using the functional localizer task [faces>scenes at *p*<0.005]), and 6-mm radius spheres were then defined around the seeds. Collectively, this approach ensured that we had bilateral fusiform cortex face-sensitive seeds for all participants, while accounting for variability in functional localization within ventral visual cortex. Because the present fMRI data were obtained using standard-resolution imaging and a relatively large smoothing kernel, this precluded finer-grained distinctions between the anatomically separate “mFus-faces” and “pFus-faces” patches of face-sensitive voxels in fusiform cortex [47]. However, the present seeds (see group-level SME “parent” seeds in Fig 3A) fell anterior to the mid-fusiform sulcus, consistent with an anatomically guided localization of the seeds to mFus-faces [50].

## Results

### Behavioral performance

#### Overall recognition memory performance

In the explicit memory task (the task of interest in the present report), participants achieved an overall hit rate of 0.71 ± 0.02 (mean ± SEM) and a FA rate of 0.29 ± 0.02, resulting in a mean *d′* of 1.20 ± 0.11. Mean response times (RTs) were faster for correct responses (hits, 1.80 ± 0.15 s; CRs, 2.10 ± 0.15 s) than for incorrect responses (misses: 2.43 ± 0.20 s; FAs, 2.30 ± 0.21 s; *t*_(18)_ = 5.15, *p* = 3.22 × 10^−5^). Hit responses were faster than CR responses (*t*_(18)_ = 5.44, *p* = 1.56 × 10^−5^).

#### Other Race Effect (ORE) in memory

Mean *d′* was significantly greater for same-race than other-race faces (same-race, 1.32 ± 0.14; other-race, 1.12 ± 0.11; ORE, *t*_(18)_ = 2.09, *p* = 0.05) (see means and distributions of *d’* for AA and EA participants in Fig 1B). While acknowledging low power, there was no evidence for a significant difference in the magnitude of the ORE between EA and AA participants (indicated by a non-significant interaction between ORE and participant race using linear mixed effects modeling to accommodate unequal sample sizes: *t*_(17)_ = 0.15, *p* = 0.88). There also was no main effect of participant race on overall memory (*t*_(17)_ = 0.06, *p* = 0.95). Due to the limited sample size for participant group-level comparisons (EA vs. AA), we refrain from analyzing participant subgroups further. Finally, there was no significant difference in mean RT for same- and other-race hits (same-race, 1.78 ± 0.14 s; other-race, 1.82 ± 0.16 s; ORE, *t*_(18)_ = 1.17, *p* = 0.26).

### Univariate fMRI analyses

We first investigated the degree to which neural activity during the encoding of same- and other-race faces was predictive of subsequent memory success. To do so, we identified subsequent memory effects (“SME”; later remembered>forgotten encoding trials), and then determined whether these encoding effects differed across same- and other-race faces.

#### Subsequent memory effects collapsing across race

Collapsing across stimulus group membership, voxel-level analysis demonstrated greater activity during successful face encoding (remembered>forgotten) in regions previously identified as associated with encoding success (for review, see [25–27,29,34]), including the left ventrolateral prefrontal cortex (inclusive of the IFS), bilateral hippocampus, and the fusiform cortex (Fig 2; Table 1).

**Table 1:** Activity SMEs collapsing across race.

	Left		Right	
	*xyz*	*t*	*xyz*	*t*
Fusiform	-44,-52,-12	5.92	40,-44,-16	5.81
Hippocampus	-28,-16,-12	4.85	28,-12,-16	4.63
Ant. Insula	-28,16,4	5.39	-	-
Suppl. Motor	-8,16,52	4.83	-	-
IFG/IFS	-52,16,28	5.42	-	-

Ant. Insula = anterior insula; Suppl. Motor = supplementary motor cortex; IFG/IFS = inferior frontal gyrus/sulcus

#### Subsequent memory Other Race Effects (OREs)–ROI analyses

Turning to the central question of interest, we conducted targeted ROI analyses of whether the subsequent memory effect (SME) varies as a function of the ORE. Using repeated-measures ANOVAs, these analyses specifically tested the hypothesis that frontoparietal components of the DAN and CCN are differentially engaged for successful vs. unsuccessful encoding of same- vs. other-race faces (i.e., SME × ORE interaction). Analyzing data from frontal (SFS and IFS) and parietal (mIPS-SPL and lIPS) components of the DAN and CCN, we first examined whether the SME (remembered vs. forgotten) varies as a function of same- vs. other-race faces, collapsing across networks (DAN vs. CCN). We then tested whether the SME varies across network (DAN vs. CCN), collapsing across OREs, and whether the effect of same-/other-race on the SME varies by network. Finally, we examined the magnitude and directionality of OREs on the SME within individual ROIs, as motivated by these analyses.

Repeated-measures ANOVAs on data from the prefrontal and parietal ROIs (Fig 3A) revealed that the SME varied as a function of the ORE, collapsing across the DAN and CCN in the left hemisphere. Specifically, the SME was greater for other-race than same-race faces in left parietal cortex, with a similar trend in left prefrontal cortex (Fig 3B; left parietal ORE on the SME: *F*_(1,18)_ = 5.13, *p* = 0.04; left prefrontal ORE on the SME: *F*_(1,18)_ = 3.06, *p* = 0.10; right parietal and prefrontal *F*s < 1). This finding supports the prediction that OREs in the SME are significantly observed in frontoparietal components of the DAN and CCN during face encoding.

The repeated-measures ANOVAs also revealed that collapsing across the ORE, there was a main effect of network on subsequent-memory dependent activity in bilateral prefrontal and right parietal DAN and CCN ROIs, reflecting a greater SME in left IFS, right SFS, and right mIPS-SPL (left/right prefrontal ROIs: *F*_(1,18)_ = 5.66/6.00, *p* = 0.03/0.02; right parietal ROI: *F*_(1,18)_ = 8.93, *p* = 0.01; left parietal *F* < 1). This finding corroborates an association between recruitment of these networks and subsequent memory success.

Finally, there were trends towards significant interactions between the effect of same-/other-race face status on the SME and network (DAN vs CCN) in the left parietal ROIs (left parietal SME by ORE × Network: *F*_(1,18)_ = 2.93, *p* = 0.10; left prefrontal SME by ORE × Network: *F*_(1,18)_ = 2.92, *p* = 0.11). Trends were not evident in right parietal and prefrontal ROIs (*F*s < 1). Collectively, these data demonstrate that the magnitude of the SME in the left parietal components of the DAN and CCN differs for same- vs. other-race faces, with trend-level differences in the ORE magnitude on the SME between the two networks. These findings support the hypothesis that the ORE in memory is related to differential recruitment of the DAN and CCN for same- vs. other-race faces.

Motivated by evidence for significant OREs on the SME in left parietal cortex (and trend-level effects in left prefrontal cortex) spanning the DAN and CCN, we conducted a follow-up analysis within individual left parietal and prefrontal ROIs. Paired *t*-tests between other-race SMEs and same-race SMEs within the left prefrontal and parietal DAN and CCN ROIs demonstrated that in the CCN activity in the parietal (and, at trend level, the prefrontal) ROI exhibited stronger SMEs for other-race than same-race faces (CCN: IFS/lIPS: *t*_(18)_ = 1.88/2.64, *p* = 0.08/0.02; DAN: SFS/mIPS-SPL: *t*_(18)_ = 1.24/1.58, *p* = 0.23/0.13) (Fig 3B). These data indicate that univariate OREs on the SME were qualitatively most robust in the CCN.

#### Subsequent memory Other Race Effects (OREs)–voxel-level analyses

Exploratory whole-brain voxel-level analysis of OREs on encoding activity revealed no significant SME differences as a function of same- vs. other-race faces. However, at a reduced cluster extent threshold (k = 29), a voxel-level difference (*p*<0.005) was observed in left SFS, falling rostral to the DAN SFS ROI (xyz = -20, 20, 60, peak *t* = 4.24), wherein the SME was larger for other- than for same-race faces. There were no significant clusters in the reverse contrast (same-race SME>other-race SME).

**Fig 4:**
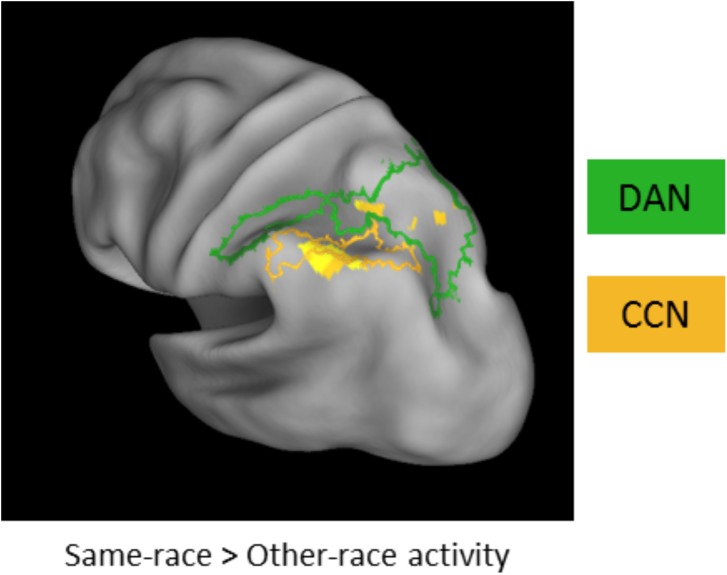
Other Race Effects on encoding activity, collapsing across memory success. Voxel-level comparison of overall activity for same- vs. other-race encoding events revealed a large cluster spanning parietal components of the DAN and CCN. Height and extent thresholds: *p* < 0.005, *k* = 33, FWE *p* < 0.05. DAN (green) and CCN (orange) ROIs overlaid for reference.

Finally, when comparing encoding-related activity regardless of subsequent memory success (following [15]), voxelwise activity in left IPS (xyz = -44, -60, 56, peak *t* = 4.58; spanning the parietal CCN and DAN ROIs; Fig 4) was significantly greater during the encoding of same- vs. other-race faces. We did not observe a similar effect of overall greater same- vs. other-race activity in fusiform cortex (collapsed across subsequent memory success) as reported by Golby et al. [15]. However, our collective parietal results suggest that such overall elevated same- vs. other-race activity during encoding is driven, in part, by reduced activity for the subset of other-race faces that were subsequently forgotten (Fig 3B).

### Functional connectivity predictive of subsequent memory

We hypothesized that OREs in memory are characterized by greater allocation of top-down attention and/or cognitive control during same- than other-race face processing at encoding. Targeted PPI ROI analyses supported this prediction, revealing that the parietal component of the DAN, in particular, showed greater memory-predictive functional covariance with fusiform cortex during the encoding of same- than other-race faces.

### Subsequent memory Other Race Effects (OREs)–PPI ROI analyses

First, repeated-measures ANOVAs (using subsequent memory-predictive fusiform connectivity computed separately for same-race and other-race face encoding) tested whether connectivity OREs manifest across the DAN and CCN. This analysis demonstrated a trend towards a significant ORE collapsing across the DAN and CCN (indicated by a marginal main effect of same-/other-race status on memory-predictive functional connectivity between right fusiform cortex and parietal ROIs (right parietal ORE on the SME PPI: *F*_(1,18)_ = 3.43, *p* = 0.08; right prefrontal ORE on the SME PPI: *F*_(1,18)_ = 1.75, *p* = 0.20). Differences were non-significant in left ROIs (left parietal ORE on the SME PPI: *F*_(1,18)_ = 2.56, *p* = 0. 13; left prefrontal ORE on the SME PPI: *F*_(1,18)_ = 0.51, *p* = 0.48). Second, collapsing across same- and other-race SMEs, there was a main effect of network on memory-predictive functional connectivity between the right fusiform cortex and prefrontal DAN and CCN ROIs, reflecting IFS-fusiform connectivity being more predictive of subsequent memory than SFS-fusiform connectivity (left/right prefrontal ROIs: *F*_(1,18)_ = 6.93/2.09, *p* = 0.02/0.17; left/right parietal ROIs: *F*_(1,18)_ = 0.81/2.58, *p* = 0.38/0.13). Finally, there were no significant interactions between the ORE and network (DAN vs CCN) in right fusiform memory-predictive connectivity (left/right prefrontal ORE on SME PPI × Network: *F*s < 1; left/right parietal ORE on SME PPI × Network: *F*s < 1) (Fig 3C), suggesting that across OREs in DAN-fusiform connectivity were not significantly larger than in CCN-fusiform connectivity.

Motivated by the observed a) significant univariate OREs on the SME in parietal cortex (Fig 3B) and b) trend-level OREs on the SME PPI with right fusiform when collapsing across networks in parietal cortex, we conducted a follow-up PPI analysis of OREs on the SME PPI within these individual ROIs (ROI × SME interaction). The results demonstrated that subsequent-memory related functional connectivity between right fusiform cortex and the parietal components of the DAN (mIPS-SPL) was significantly greater for same- than other-race faces (left mIPS-SPL: *t*_(18)_ = 2.45, *p* = 0.02; right mIPS-SPL: *t*_(18)_ = 2.07, *p* = 0.05); significant effects were not observed in the parietal components of the CCN (left lIPS: *t*_(18)_ = 1.00, *p* = 0.33; right lIPS: *t*_(18)_ = 1.44, *p* = 0.17). Thus, in contrast to the univariate activity findings reported above, the PPI OREs on memory-related connectivity were qualitatively more robust in the DAN.

The left fusiform seed did not show the same PPI pattern as right fusiform, in that there were no significant OREs on memory-predictive connectivity (all parietal and prefrontal *F*s < 1). However, there was a main effect of network on memory-predictive connectivity between the left fusiform cortex and prefrontal and parietal DAN and CCN ROIs, reflecting stronger memory-related connectivity with IFS and (in the right hemisphere) with lateral IPS than with SFS and mIPS-SPL (left/right hemisphere prefrontal ROIs: *F*_(1,18)_ = 17.06/6.59, *p* = 6.28x10^-4^/0.02; left/right hemisphere parietal ROIs: *F*_(1,18)_ = 3.00/6.58, *p* = 0.10/0.02). There were no significant interactions between ORE on functional connectivity and network (DAN vs CCN) in left fusiform connectivity (all *F*s < 1), and no ORE × SME PPI interactions within individual ROIs approached significance (all *t*s < = 1).

### Subsequent memory Other Race Effects (OREs)–PPI voxel-level analyses

**Fig 5:**
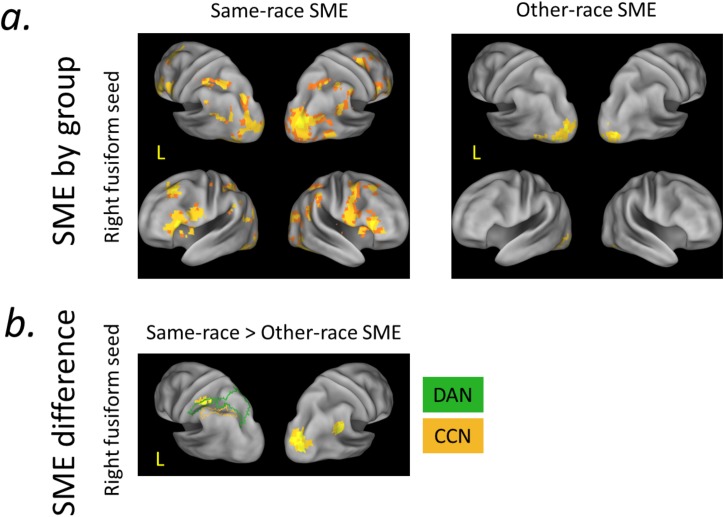
Voxel-level subsequent-memory predictive fusiform functional connectivity. a) Same-race face encoding success was predicted by widespread functional interactions between fusiform cortex and cortical networks associated with top-down attention and cognitive control. Other-race face encoding did not show similar memory-related functional interactions with right fusiform cortex. b) Consistent with the ROI-based analysis, left IPS showed significantly greater subsequent-memory predictive fusiform connectivity for same- than other-race faces. DAN (green) and CCN (orange) ROIs overlaid for reference. PPI effects rendered as described in Fig 2; height and extent thresholds: *p* < 0.005, *k* = 33, FWE *p* < 0.05.

To complement the aforementioned ROI analyses, we next conducted a voxel-level PPI analysis of subsequent memory-predictive fusiform connectivity separately for same-race and other-race face encoding, and then examined the interaction between same-/other-race status and SME (ORE × SME on fusiform connectivity). Voxel-level analysis of PPIs with right fusiform cortex revealed prominent memory-related functional interactions with prefrontal and parietal voxels that fell in the DAN and, to a lesser extent, CCN for same-race faces (Fig 5A, left panel; Table 2). By contrast, right fusiform memory-related functional connectivity with prefrontal and parietal voxels was not significant for other-races faces (Fig 5A, right panel). Importantly, a significant voxelwise ORE (same-race>other-race SME) was observed in connectivity between right fusiform and the rostral extent of left IPS, which corresponds to the anterior-most portion of the parietal DAN component (consistent with the results of the above reported ROI analysis; Fig 5B). Left fusiform cortex did not exhibit marked differences in memory-related connectivity for same vs. other-race faces (Tables 3 and 4). There were no instances of the reverse pattern, wherein the SME PPI with fusiform showed an other-race>same-race effect.

**Table 2:** PPI SMEs by stimulus race. Connectivity with right fusiform seed.

**Same-race**				
	Left		Right	
	*xyz*	*t*	*xyz*	*t*
Fusiform	-36,-52,-24	6.87	36,-56,-16	5.95
MFG	-32, 20, 44	6.23	36,20,48	4.87
IFG/IFS	-48,28,4	5.66	60,24,16	4.04
Lat. Occip.	-24,-92,12	3.93	36,-88,16	6.04
IPS (medial peak)	-28,-48,48	4.85	28,-52,48	4.21
SMG	-52,-44, 36	3.67	56,-36,32	3.76
Post. Cing.	-4,-32,40	4.30	-	-
Putamen	-20,8,4	3.20	20,16,4	3.48
**Other-race**				
	Left		Right	
	*xyz*	*t*	*xyz*	*t*
Fusiform	-36,-68,-8	5.80	36,-64,-12	5.13
Lat. Occip.	-28,-88,8	5.68	28,-84,0	6.13

MFG = middle frontal gyrus; IFG/IFS = inferior frontal gyrus/sulcus; Lat. Occip. = lateral occipital cortex; IPS = intraparietal sulcus; SMG = supramarginal gyrus; Post. Cing. = posterior cingulate

**Table 3:** PPI SMEs by stimulus race. Connectivity with left fusiform seed.

**Same-race**				
	Left		Right	
	*xyz*	*t*	*xyz*	*t*
Fusiform	-40,-64,-16	5.71	32,-64,-20	6.12
Ant. Insula	-32,12,0	4.72	32,8,4	4.58
MFG	-32,36,36	4.76	44,4,40	6.40
IFG/IFS	-44,8,12	6.50	56,4,20	5.00
Lat. Occip.	-28,-84,8	4.24	32,-84,12	6.22
Post. Cing.	-	-	8, -24, 32	4.85
IPS (medial peak)	-32,-48,44	4.97	36,-44,48	4.73
SMG	-64,-28,36	3.73	56,-32,40	4.58
Angular gyrus	-	-	56,-48,28	5.17
Caudate	-12,8,16	4.78	16,16,8	4.39
Putamen	-28,12,8	5.43	-	-
**Other-race**				
	Left		Right	
	*xyz*	*t*	*xyz*	*t*
Fusiform	-40,-48,-28	5.73	-40,-56,-12	4.65
Ant. Insula	-36,12,4	5.60	40,4,0	3.96
IFG/IFS	-48,8,16	5.28	44,4,32	4.17
IPS	-28,-52,48 (medial)	3.71	40,-44,42 (lateral)	4.00
Lat. Occip.	-28,-88,4	4.97	40,-88,0	3.52
Putamen	-28,8,4	2.98	24,16,4	3.76

Ant. Insula = anterior insula; MFG = middle frontal gyrus; IFG/IFS = inferior frontal gyrus/sulcus;

Lat. Occip. = lateral occipital cortex; Post. Cing. = posterior cingulate; IPS = intraparietal sulcus;

SMG = supramarginal gyrus

**Table 4:** PPI OREs on the SME.

**left Fus. seed, Same-race>Other-race**				
	Left		Right	
	*xyz*	*t*	*xyz*	*t*
-	-	-	-	-
**right Fus. seed, Same-race>Other-race**				
	Left		Right	
	*xyz*	*t*	*xyz*	*t*
SMG	-	-	52,-48,28	4.79
IPS (medial peak)	-40,-32,48	4.82	-	-
Lat. Occip.	-	-	28,-88,16	4.62

SMG = supramarginal gyrus; IPS = intraparietal sulcus; Lat. Occip. = lateral occipital cortex. Fus. seed = fusiform seed

## Discussion

The present study provides a novel demonstration that successful encoding of same- and other-race faces is differentially predicted by engagement of top-down visuospatial attention and cognitive control frontoparietal networks. Specifically, our data show that parietal components of the CCN and DAN exhibited significantly different levels of subsequent memory-related encoding activity and fusiform functional connectivity, respectively, for same-race and other-race faces. Strikingly, the analyses of univariate activity demonstrate that reduced engagement of the parietal component of the CCN is predictive of memory *failure* for other-race faces, consistent with our prediction that the encoding of faces from another race is generally subjected to a more superficial level of processing. Conversely, the results of the PPI analyses demonstrate that interactions between the parietal component of the top-down DAN and fusiform cortex is more strongly predictive of memory *success* for same-race faces, consistent with our prediction that attention is differentially allocated to faces of one’s own group in a manner which facilitates memory encoding.

In addition to the ORE on encoding activity, wherein univariate activity was differentially reduced in the parietal component of the CCN during the encoding of other-race faces that were later forgotten, we observed robust differences in the degree to which the DAN and CCN contribute to subsequent memory when collapsing across stimulus race (Figs 2 and 3). Specifically, the engagement of the left VLPFC/IFS was generally more predictive of memory success in our elaborative intentional encoding task than was left SFS. However, network level differences between the DAN and CCN in the ORE on the SME did not exceed trend level. Collectively, these analyses of univariate activity, combined with the PPI analyses of memory-related functional interactions with fusiform cortex, support a framework in which the cognitive functions supported by both the DAN and CCN contribute to OREs observed in memory behavior.

Recruitment of the DAN and CCN frontoparietal networks is well established to influence episodic encoding success, as indexed by subsequent memory performance [25–30], while OREs in memory have been previously associated with greater activity in face-sensitive areas of fusiform cortex during the processing of same- vs. other-race faces [15–17]. While differences in fusiform processing may arise from the region’s sensitivity to perceptual expertise [15,18,19], it has been proposed that cognitive and attentional modulation during encoding may contribute to OREs in neural activity and memory behavior [15,20,21,24]. Our findings lend novel support to this possibility, demonstrating that the ORE in subsequent memory is predicted by differential engagement of attention and cognitive control circuitry during the encoding of same- and other-race faces. Frontoparietal circuitry also supports visual working memory, which could interact with and facilitate top-down attention and cognitive control operations [51] and contribute to elaborative encoding in our task. Event-related potentials associated with visual working memory reflect processing differences for same- vs other-race face identities [52,53]. Such data suggest that differences in the quality of face representations in active maintenance during encoding could contribute to subsequent memory success. It will be of particular interest in future research to design paradigms aimed at dissociating the contributions of working memory, attention, and cognitive control processes to race effects in long-term memory encoding.

Recent work has demonstrated that top-down factors such as processing goals can exert a powerful influence over how same- vs. other-race faces are processed, potentially attenuating or exacerbating group biases [20,21]. For example, when the race of face stimuli is orthogonal to alternative, task-relevant group categories (e.g., sports teams), activation differences in fusiform cortex shift from same-*race* to same-*group* [35–37], a shift that is also observed in behavior (e.g., in automatic face preference and valence evaluations and in memory biases; [36,38]). Similarly, when biracial individuals are oriented to one or the other of their racial backgrounds, OREs in perception and self-stereotyping shift according to the race to which they are oriented [54,55]. Importantly, the discriminability of fusiform representations of faces from different races is influenced by the severity of racial bias [56] as well as processing goals such as the type of social group categorization task being performed [45]. Collectively, such data suggest that group membership categories and orientations can change how attention and control processes are allocated during face processing, which can influence how faces are represented. Group categorization effects can affect the perceptual processing of faces within a few hundred milliseconds [3,4]. Moreover, people exhibit a tendency towards more rapid and accurate categorization of faces of another race as being of another race than faces of an individual’s own race as being same-race [5–11]. One possibility is that rapid group categorization effects may contribute “downstream” to how top-down attention and cognitive control are deployed–that is, the rapid identification of a face’s group membership (and thus potentially its salience) could influence how deeply the stimulus is processed, which could in turn influence its successful encoding in memory. Recent work has shown increased functional engagement of prefrontal cortex (including inferior frontal gyrus) with fusiform cortex when categorizing faces as same-race than other-race [6]. Our data demonstrate that when people engage in intentional, elaborative encoding of face stimuli, fusiform processing comes under top-down attentional modulation that is more predictive of successful same-race than other-race encoding. The tendency for more robust effects involving right fusiform cortex may support a lateralization in memory-predictive face processing, and could relate to findings linking right fusiform face area to processing familiarity in faces [57].

Our univariate analysis revealed that people generally recruited parietal components of both the DAN and CCN more strongly for same-race than other-race encoding (Fig 4). This finding is broadly consistent with the involvement of these frontoparietal networks in successful encoding processes [25–30], and a framework in which greater cognitive resources are directed towards faces perceived as sharing one’s group membership [15]. However, whereas functional interactions between the DAN and fusiform cortex more strongly related to same- than other-race subsequent memory success, greater overall univariate activity for same-race faces in the IPS appeared to be driven by greater other-race SMEs. Specifically, reduced engagement of the cognitive control network was more predictive of memory failure for other-race faces. These findings may be complementary to the same-race bias in attention interactions with fusiform processing, suggesting that the encoding of faces from another race is generally subjected to a more superficial level of processing.

It is possible that retrieval-related processes also contribute to our observed encoding effects. Specifically, given that the intentional encoding task encouraged participants to generate a story around each face, successful same-race encoding (or, conversely, less successful other-race encoding) could be influenced strongly by the ability to generate stories from prior experiences that contain faces in them, or even retrieving memories associated with perceptually similar faces. To the extent that people may have less familiarity with stories and social contexts involving other-race individuals, our intentional encoding task demands could expose a way in which differences in mnemonic experience can help give rise to OREs during encoding. Within such a framework, greater top-down attention and cognitive control could contribute to generating more differentiated representations at encoding that give rise to better memory discrimination later–this relationship to subsequent memory may further reflect the contributions these processes could make to the ability to place faces within a narrative context (whether retrieved or constructed). The contributions of posterior parietal cortex to episodic retrieval have become an increasingly active area of research [58,59], and one intriguing possibility is that the parietal components of the top-down attention and cognitive control networks contribute to deeper narrative processing for same-race faces in addition to influencing the necessary item-level encoding within face-sensitive cortex. Such processing biases may, ultimately, provide a mechanistic framework for better memory for the motivationally relevant faces of individuals from social groups with which we identify. While many social encounters involve intentional encoding of the identity of the individual we have met, one critical question to address in future research is whether the same mechanisms associated with the ORE in our paradigm extend to cases where encoding of faces is incidental, as may occur in other facets of daily life.

One limitation of the present study is the impact of a sample size appropriate for our within-participants analyses on the ability to characterize individual differences in brain activity and behavior. For example, it will be of interest in future work to test whether the neural correlates of OREs in subsequent memory, as examined in the present study, relate to differences in how well individuals are able to encode and recognize other-race faces. Specifically, one question to be addressed is whether the relationship between cognitive control engagement and other-race memory failure is stronger in individuals with greater same-race memory biases, and whether these biases relate to measures of perceptual expertise and/or the strength with which people identify with their racial group. Stable, individual differences in implicit bias could affect attention to same- and other-race faces, and these could combine with or counteract situational factors that influence the relevance of race and other group identity cues. It is important to note that the temporal resolution of fMRI leaves open the possibility that top-down influences on fusiform face processing follow rapid initial perceptual expertise-based processing differences. Similarly, our modest sample size was unbalanced in terms of the number of EA and AA participants, limiting our ability to test for potential race differences in neural OREs. There is evidence, for example, that EA and AA individuals share a bias in amygdala activity towards AA faces at encoding [16], suggesting that mutual cultural associations and other factors may influence the magnitude or directionality of neural OREs for different racial groups. Experiments designed for effective connectivity measures [60] could also prove informative, providing further insight into the directional relationship between fusiform face processing and frontoparietal networks. Moving forward, it will be of interest to conduct large-sample and high-trial count experiments that address such questions and further our understanding of the factors that modulate racial biases in cognition.

## Conclusion

Understanding the neural mechanisms that underlie and influence relations between different social groups is of central importance for society. Research has long suggested that social category information, such as race and gender, is automatically encoded [1,2], and that social categories can influence cognition, with implications for behavior in social and legal settings. Our findings provide novel evidence that the tendency for individuals to exhibit better recognition memory for faces from their own race (or, potentially, social group) relates to differential allocation of top-down attention and cognitive control resources. Failure to remember other-race faces may arise in part due to reduced engagement of cognitive control mechanisms underlying deeper, elaborative encoding, while successful memory for same-race faces is facilitated by increased functional engagement of top-down attention during face processing. Collectively, these data advance understanding of the neural mechanisms that underlie racial biases in memory.
